# Clinical Evidence of Wear Occurrence in CFR-PEEK and Metallic Osteosynthesis Implants: A Systematic Literature Review

**DOI:** 10.3390/bioengineering12090965

**Published:** 2025-09-08

**Authors:** Remco Doodkorte, Rachèl Kuske, Jacobus Arts

**Affiliations:** 1 Department of Orthopaedic Surgery, CAPHRI Research School, Maastricht University Medical Center (MUMC+), P. Debyelaan 25, 6229 ET Maastricht, The Netherlands; j.arts@mumc.nl; 2 Orthopaedic Biomechanics, Department of Biomedical Engineering, Eindhoven University of Technology, Theodor Fliednerstraat 2, 5631 BN Eindhoven, The Netherlands; rkuske@live.nl

**Keywords:** CFR-PEEK, metallic, osteosynthesis, open reduction and internal fixation, intramedullary nailing, wear, debris

## Abstract

Carbon fiber-reinforced polyetheretherketone (CFR-PEEK) as an alternative to metallics in orthopedic implants offers biomechanical and radiological advantages. However, the extent of wear particle generation and its clinical impact are unclear. This systematic review evaluates clinical evidence of wear in fracture fixation devices. A systematic search was conducted to identify clinical studies reporting wear of metallic and CFR-PEEK implants used in extremities. Nineteen studies were included: three prospective cohorts, eight retrospective cohorts, one case series, and six case reports. Among 208 fixation plates, 43 were CFR-PEEK and all 93 intramedullary nails were metallic. Risk of bias ranged from low to serious, mainly due to selection bias. Wear-related complications were reported for both materials. Metallic implants showed elevated serum ion levels, metallic debris in tissues, and, in some cases, metallosis. CFR-PEEK implants showed limited evidence of carbon fiber fragments near implants. One comparative study reported higher inflammatory responses in CFR-PEEK explants, though no direct link between debris and implant removal was found. Both metallic and CFR-PEEK fracture fixation devices generate wear particles, which may induce biological responses. However, wear-related complications appear rare, especially with validated implant designs, and clinical significance of wear debris remains limited.

## 1. Introduction

Fracture fixation devices play a crucial role in orthopedic treatment by ensuring mechanical stability, bone (re-)alignment, and early mobilization, thus promoting efficient recovery. The choice of stabilization method is determined by fracture type and severity. Despite technological advancements, revision rates are still as high as 14.8% and 6.8% for open reduction and internal fixation (ORIF) and intramedullary nailing, respectively [1,2]. One contributing factor may be implant wear, which has been extensively studied in metal-on-metal joint arthroplasty but remains less explored in fracture fixation.

For fracture fixation devices, material selection plays a critical role in long-term success, as wear-induced implant degradation may contribute to failure. Metals, which have long been the standard in orthopedic implants, have been linked to wear-related complications [3]. Metal-induced osteolysis is a concern since wear debris from materials like cobalt, chromium, titanium (Ti), or stainless steel (SS) can elicit local tissue reactions and systemic effects [4]. Histological analyses of failed implants frequently reveal metallosis and lymphocytic infiltration in periprosthetic tissues [5]. This underscores the need for improved materials to better resist wear and mitigate adverse biological responses, paving the way for alternative solutions.

Given these concerns, alternative materials such as carbon fiber-reinforced polyetheretherketone (CFR-PEEK) have gained attention for their potential to reduce wear-related complications. This composite material combines long continuous carbon fibers within a matrix of PEEK polymer, offering tailorable mechanical properties and biological benefits. Notably, by altering the orientation of the carbon fibers, the elastic modulus of CFR-PEEK can be adjusted, ranging from near Ti alloy to human cortical bone [6]. In contrast, traditional metallic materials such as Ti (110–120 GPa) [7] and SS (193 GPa) [8] have a higher elastic modulus. This ability to reduce stiffness while maintaining strength reduces stress shielding, enabling more effective load sharing and promoting bone healing [9]. In addition to its mechanical properties, CFR-PEEK is recognized for its high strength and resistance to deformation, making it a durable material for fracture fixation [10]. Furthermore, its excellent chemical stability ensures long-term durability [11], while its radiolucency allows for clear post-operative imaging by reducing artefacts in MRI and CT scans. CFR-PEEK also demonstrates superior resistance to fatigue failure [6].

These distinctive mechanical, chemical, and radiological properties make CFR-PEEK a highly promising biomaterial, offering several advantages over traditional metallic implants for fracture fixation applications. Despite CFR-PEEK’s advantages, the extent to which wear particle generation poses a clinical concern remains unclear. Furthermore, it is of interest how this potential wear compares to that of metallic implants. To address that gap, this systematic review aims to evaluate clinical evidence of wear in fracture fixation devices, comparing metallic and CFR-PEEK implants.

## 2. Materials and Methods

### 2.1. Search Strategy

This study was performed in accordance with the Preferred Reporting Items for Systematic Review and Meta-Analyses guidelines (Table S1: PRISMA checklist) [12]. Two separate search strategies were developed to identify all relevant records for clinical evidence of, firstly, wear in fracture fixation in general, and secondly, all journal articles making any reference to CFR-PEEK. The latter was specifically developed to avoid underreporting of wear using CFR-PEEK implants. A detailed search strategy can be found by consulting Appendix A.

### 2.2. Eligibility Criteria

All clinical reports were eligible for inclusion. Studies were included if they involved patients treated for traumatic and or pathological fractures of the long bones, hands, and feet using plates and or intramedullary nails (IMNs). No language restrictions impeded on the study selection.

### 2.3. Study Selection and Critical Appraisal

**Figure 1:**
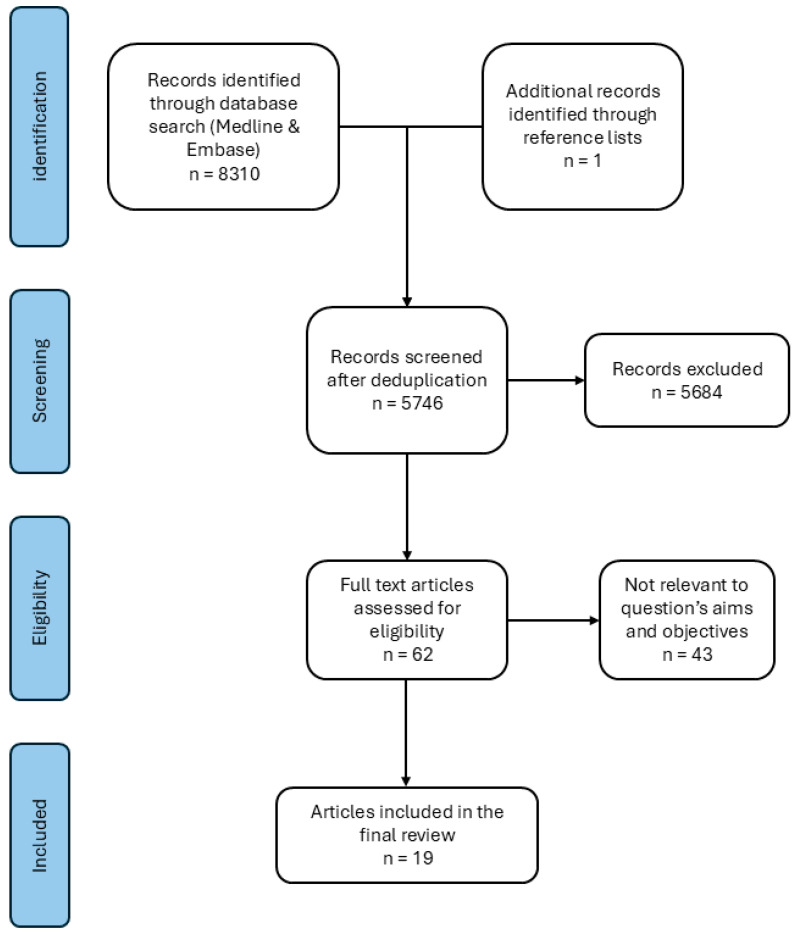
PRISMA flow diagram of study selection.

Medline and Embase databases were screened, with the search performed on 1 March 2025. Search results were limited to papers after 1 January 2000. After retrieval of eligible studies and deduplication, title and abstract screening was performed on 5746 articles (Figure 1). Full-text screening was performed on 62 articles, of which 18 studies were included in this systematic review. One additional article was included from the reference lists. In case of trauma, studies were only included if osteosynthesis material had remained in situ >6 months after a traumatic event. Included articles were screened using the Risk of Bias in Non-Randomized Studies of Interventions tool (ROBINS-I version 2;2024) [13]. Authors RD and RK performed the article screening and quality assessment, and SG was consulted in case of any disagreement.

### 2.4. Data Extraction

A standardized extraction form was used to extra the data. The data captured included study characteristics (authors, year, type of study), patient characteristics (patients, implanted material, anatomical location, and evidence of wear including incidence and serum ion measurements) and additional findings. Data extraction was performed between plating and intramedullary nailing.

## 3. Results

### 3.1. Study Characteristics and Critical Appraisal

A total of 19 studies involving a total of 301 implants were included. The patients were included in three prospective cohort studies [14,15,16], eight retrospective cohort studies [17,18,19,20,21,22,23,24], one case series [25], and six individual case studies [5,26,27,28,29,30,31]. These studies included a total of 208 fixation plates, of which 43 were made out of CFR-PEEK. The other plates were made of Ti, SS, or an unspecified metallic alloy. With regard to the 93 IMNs, no CFR-PEEK nails were included.

The risk of bias assessment of non-randomized studies ranged from low (n = 5) to serious (n = 5), with a single study having moderate risk of bias (Table 1). Serious risk of bias was primarily due to selection bias. The case series scored 6/8 or higher, with all perfect scores for describing the clinical condition, interventions, and assessment method (Table 2). Overall, due to the limited level of evidence due to the absence of randomized controlled trials and the presented risks of bias, the results should be interpreted with caution.

### 3.2. Reported Wear in Plate Fixation

Thirteen studies investigated the presence of wear of metallic plating material, which included Ti, SS, and implants of unspecified material composition. The anatomical sites included the femur, humerus, tibia, fibula, radius, metacarpals, radius, and clavicle. One of these studies directly compared to CFR-PEEK implants [24]. One additional case study and one retrospective cohort study investigated wear of CFR-PEEK in a radius plate and tibial plates, respectively [17,27]. Although the latter was a matched pair comparison with Ti plates, analysis to identify wear particles was only performed on the CFR-PEEK population, and hence the other population was not considered (Table 3).

Only a single study directly compared CFR-PEEK to metallic fracture fixation plates [24]. In this retrospective cohort study, the inflammatory response and implant surfaces were analyzed after explantation due to impaired movement and/or persistent pain. All peri-implant tissues were positive for inflammatory macrophages, which was significantly higher for CFR-PEEK samples. Also, all peri-implant tissues were positive for either CFR-PEEK or Ti particles. Notably, the tantalum wires incorporated for their radiopacity were shown to protrude into the screw holes. Similar results were seen in the two other studies investigating CFR-PEEK implants. In the study by Cotic et al., 15/26 (58%) tissue samples revealed the presence of foreign body material although no sign of acute inflammation was present [17]. The case study of a distal radius plate showed synovitis at the implant site, and histopathology showed the carbon fibers in multinucleated cells [27]. Nevertheless, the implant surface did not show clear signs of wear, neither at the flat implant surface nor at the screw threads.

Besides the comparison study described above, an additional five studies investigated the presence of metallic plate fixation-derived wear debris in cohort studies [15,18,19,20,21]. In the largest retrospective study by Park et al., 69 plate removals after 1 year of implantation were studied, in which wear debris-induced metallosis occurred in 4/38 (5.8%) cases using a specialized fibular locking plate, whereas no metallosis was observed in the patients treated with other fibular ORIF devices [19]. In a similar retrospective study with the same plates (Zimmer^®^ ZPLP, Zimmer Biomet, Warsaw, IN, USA) by Yeo et al., five cases of wear-induced metallosis were diagnosed in a cohort of 27 fracture fixations (18.5%) [21]. Krischak et al. performed a study of 22 plate removals at various anatomical locations, for which complications were the indication of removal [18]. In total, 5/8 (62.5%) Ti implants and 14/14 (100%) of the SS showed some degree of surface corrosion. The majority of the biopsies from these patients confirmed the presence of wear debris through histological analysis of biopsies. The final retrospective study involved hip-screw plates [20], in which each of the explanted plates showed clear wear around the lag screws. Notably, one prospective study was identified [15]. Despite the absence of histological analysis, impairing conclusions of wear, significantly increased serum ion levels were present 24 weeks after implantation (n = 10).

The other identified papers studying wear in plate fixation describe case studies/series. Bertoldi describes a case series of 18 implants with unknown anatomical locations, revealing the presence of metallic debris particles in surrounding tissues, for which the level of inflammation was related to the amount of Ti particles [25]. The remaining case studies each present clear wear and or metallosis due to debris, which, for each of the cases, can be related to some kind of implant failure. The causes for wear were two separate plates rubbed against each other [29], wear of separate plate segments after failure [26], and friction-induced particles related to non-union [5,28].

### 3.3. Reported Wear of IMN for Fracture Fixation

Reporting of wear in IMNs was identified in seven studies, of which one also involved fixation plates [15] (Table 4). Serum measurements from patients with IMNs in the femur (n = 4) or tibia (n = 6) showed significantly elevated serum ion levels after 24 weeks. Comparably, significantly increased Ti or chromium serum ion levels were detected in a total of 41 IMNs [14]. Interestingly, elevated Ti serum ion levels were only detected in 25 modular IMNs by Jones et al. [16]. Additional histopathology and scanning electron microscopy identified corrosion at the modular junction.

In agreement with Jones et al., the two retrospective cohort studies and two case studies demonstrated wear in multi-component IMNs [16]. Foong inspected 11 retrieved femur or tibia-lengthening nails, showing notches in the nails at regular intervals corresponding to the limb-lengthening intervals [22]. Similar to the lag screw plates, proximal femoral nails with an intramedullary femoral neck element show regular scratch marks in case of migration [23]. Finally, Ngo et al. and Kang et al. identified wear in single cases of intramedullary nailing through a telescopic femoral nail and a flexible humeral nail, respectively [30,31].

## 4. Discussion

The development of wear debris in total joint arthroplasty may have severe consequences and thus this topic has been extensively studied for metallic implants [32], though evidence for CFR-PEEK bearing surfaces remains less conclusive [33]. The presence and biological effects of wear particles, either metallic or CFR-PEEK, has been briefly studied in osteosynthesis. Therefore, the goal of this study was to identify and compare the existing literature providing clinical evidence of wear after osteosynthesis using either metallic or CFR-PEEK implants. Although a total of 19 studies were included, the level of evidence was generally low, with various degrees of risk of bias. Based on these studies, a few trends can be identified and further discussed. Wear particles are associated with the need for revision in rare cases of trauma, unique cases of surgical anomalies, or the use of implants later recalled due to design flaws. The general presence of wear particles can be found surrounding Ti, SS, and CFR-PEEK implants. Hence, conclusions based on this literature study should be drawn with caution.

Firstly, as mentioned, the level of evidence is very limited. Although multiple authors have attempted to develop prospective studies in which the effect of debris is measured systemically through serum ion levels, this is an indirect measure of wear. Nevertheless, Jones et al. did show corrosion on the explanted implant related to higher serum levels [16]. The remaining evidence is from retrievals in retrospective studies or individual cases which have an inherent risk of selection bias. Moreover, study populations were small in general, providing a wear rate varying from 5.8 to 100%. This selection bias is further enhanced by the fact that removal of osteosynthesis material without relevant cause such as implant failure or irritation will not be reported at all.

Nevertheless, there are studies reporting wear, which in turn provide us with an insight into the question on whether wear is matter of concern in osteosynthesis with fracture plates and IMNS or not. Similar to the metal-on-metal wear seen in hip arthroplasties, all individual case studies of IMNs [22,23,30,31] and plates [5,20,26,28,29] demonstrate the possible complications in case of metal-on-metal articulation. Also, the case study on a fractured CFR-PEEK radius plate resulted in flexor tendon synovitis [27]; however, this is likely caused by tendon irritation rather than an inflammatory response to debris particles. The effect of implant material on the possible development of wear debris can therefore not be concluded from these studies as they can all be categorized as implant failure or design flaws ultimately resulting in implant recall. Implant recall was also initiated for the Zimmer^®^ ZLZP plating system due to improper mating of the locking screws and threads. Considering the identified scientific evidence, the studies by Park et al. [19] and Yeo et al. [21] involved this particular series of implants, further impeding conclusions. This stresses that careful selection of material combinations and subsequent testing is needed for well-functioning implant designs.

Although sparse, there is evidence of material loss and/or the presence of debris either systemic or in the implant surrounding tissue. Multiple studies found significant amounts of metallic serum ions (Ti, Cr, Al, V, and Cr). Systemic ions have been associated with corrosion at metal-on-metal junctions [34]; however, the clinical impact on these levels and the association with osteolysis have not been defined. Furthermore, all studies that biopsied the peri-implant tissues showed either visually clear discoloration or histopathological proof of the presence of particles. This holds true for both metallic and CFR-PEEK implants. Further investigation into these particles by Fleischhacker et al. [24] demonstrated an inflammatory tissue response, resulting in macrophage activity for both Ti and CFR-PEEK implants. It must be noted that these effects were significantly higher for the CFR-PEEK implants, while the authors speculated about this being caused by exposed tantalum wires in the threaded screw holes, stressing the importance of implant design. Moreover, long-term results of a CFR-PEEK rotating hinge joint provide evidence that the inflammatory response does not result in clinically relevant complications [35]. So, implant wear material and ions are found in the peri-implant tissue and systemically; however, the direct relation between these findings and the need for implant removal has not been demonstrated.

This systematic review has provided clear insights into the prevalence and measure of debris in osteosynthesis material for the extremities. Multiple studies have shown the clear presence of particulate matter in the peri-implant materials, wear marks on modular implants, and a systemic presence of metallic ions. However, the limited amount and level of scientific evidence suggest that clinical effects of wear debris in either metallic or CFR-PEEK osteosynthesis material are rare, especially in case of properly verified implant designs.

## Figures and Tables

**Table 1:** Critical appraisal risk of bias analysis for non-randomized studies (ROBINS-I).

	Confounding	Classification	Selection	Deviation	Missing Data	Outcome Measures	Reporting	Overall
Cotic	Low	Low	Low	Low	Low	Low	Low	Low
Fleschhacker	Low	Low	Serious	Low	Low	Moderate	Low	Serious
Krischak	Low	Low	Low	Low	Low	Moderate	Low	Moderate
Park	Low	Low	Serious	Low	Low	Moderate	Low	Serious
Tanoğlu	Low	Low	Low	Low	Low	Low	Low	Low
Shahgaldi	Low	Low	Serious	Low	Low	Low	Low	Serious
Yeo	Low	Low	Low	Low	Low	Low	Low	Low
Foong	Low	Low	Low	Low	Low	Low	Low	Low
Patton	Low	Low	Low	Low	Low	Serious	Low	Serious
Jones	Low	Low	Low	Low	Low	Low	Low	Low
Law	Low	Low	serious	Low	Low	Low	Low	Serious

**Table 2:** Critical appraisal checklist from Joanna Briggs Institute for case reports.

JBI Checklist Questions	Bertoldi	Edelstein	Kumar	Merolli	Metikala	Tang	Kang	Ngo
1. Were the patient’s demographic characteristics clearly described?	N	Y	Y	Y	Y	Y	Y	Y
2. Was the patient’s history clearly described and presented as a timeline?	N	Y	Y	Y	Y	Y	Y	Y
3. Was the current clinical condition of the patient on presentation clearly described?	Y	Y	Y	Y	Y	Y	Y	Y
4. Were diagnostic tests or assessment methods and the results clearly described?	Y	Y	Y	Y	Y	Y	Y	Y
5. Was the intervention(s) or treatment procedure(s) clearly described?	Y	Y	Y	Y	Y	Y	Y	Y
6. Was the post-intervention clinical condition clearly described?	Y	NA	Y	U	Y	Y	Y	Y
7. Were adverse events (harms) or unanticipated events identified and described?	Y	N	Y	Y	Y	Y	Y	Y
8. Does the case report provide takeaway lessons?	Y	Y	Y	Y	Y	Y	Y	Y

**Table 3:** Study characteristics and outcomes of included studies involving osteosynthesis plates.

Article	Material	n	Anatomical Site(s)	Wear Incidence/Indication
[24]	CFR-PEEK Ti	16	Humerus	CFR-PEEK: 8/8 (100%) Ti: 8/8 (100%)
[29]	Ti	1	Fourth and fifth metacarpals	1/1 (100%)
[19]	SS	69	Fibula	4/69 (5.8%)
[26]	Metallic	1	Femur	1/1 (100%) Preceding trauma 6 months prior to examination
[15] ^†^	Ti	10	Femur (4 plate) Tibia (6 plate)	Significant increase in serum Ti, Al, V, and Mo ions
[27]	CFR-PEEK	1	Radius	1/1 (100%)
[17]	CFR-PEEK	26	Tibia	CFR-PEEK: 15/26 (58%)
[28]	Ti	1	Humerus	1/1 (100%)
[21]	SS	27	Fibula	5/27 (18.5%)
[5]	Metallic	1	Humerus	1/1 (100%)
[18]	Ti SS	22	Ti: fibula (4), clavicula (1), radius (3) SS: fibula (11), tibia (1), radius (2)	Ti: 5/8 (62.5%) SS: 14/14 (100%)
[25]	Metallic	18	Unknown	
[20]	SS	15	Femur	Wear identified on the screws and barrels

^†^ Also in IMN table. Only plate cases presented in this table, Ti = titanium, SS = stainless steel, Al = aluminum, V = Vanadium, Mo = molybdenum.

**Table 4:** Study characteristics and outcomes of included studies involving intramedullary nails (IMNs).

First Author	Material	n	Anatomical Site(s)	Wear Incidence/Indication
[31]	SS	1	Femur	1/1 (100%)
[23]	Ti	4	Femur	2/4 (50%)
[15] ^†^	Ti	10	Femur (4) Tibia (6)	Significant increase in the serum Ti, Al, V, and Mo ion levels
[22]	Ti	11	Femur	11/11 (100%)
[14]	Metallic	41	Tibia	Significant increase in the serum Ti and Cr
[30]	Ti	1	Humerus	1/1 (100%)
[16]	SS	25	Femur	Increased Cr levels in modular IMN (1.04 ± 0.57 ng/mL) compared to one-piece IMNs (0.26 ± 0.40 ng/mL) or control (0.05 0.06 ng/mL)

^†^ Also in plates table. Only IMN cases presented in this table. Ti = titanium, SS = stainless steel, Al = aluminum, V = vanadium, Mo = molybdenum, Cr = chromium.

## Supplementary Materials

The following supporting information can be downloaded at: https://www.mdpi.com/article/10.3390/bioengineering12090965/s1, Table S1: PRISMA checklist.

## Abbreviations

The following abbreviations are used in this manuscript:

ORIF	Open reduction and internal fixation
SS	Stainless steel
CFR	Carbon fiber-reinforced
PEEK	Polyetheretherketone
IMN	Intramedullary nail

## Appendix A. Systematic Search Terms

(Metallosis OR metalosis OR wear OR debris)
AND
(ORIF OR open reduction and internal fixation OR IRF OR internal rigid fixation OR break OR nonunion OR non-union OR malunion OR fractur* OR osteosynthesis)
OR
(carbon fiber reinforced poly* OR carbon fibre reinforced poly* OR carbon fiber reinforced poly ether* OR carbon fibre reinforced poly ether* OR Carbon Fiber Reinforced PEEK OR Carbon Fibre Reinforced PEEK OR CFR PEEK OR CFRPEEK OR CF/PEEK OR Carbon PEEK OR Carbon fiber plate* OR Carbon fibre plate* OR CF plate* OR CFR plate* OR Carbon plate*OR PEEK plate OR radiolucent plate* OR Carbon fiber implant* OR Carbon fibre implant* OR CF implant* OR CFR implant* OR Carbon implant*OR PEEK implant* OR radiolucent implant* OR Carbon fiber nail* OR Carbon fibre nail* OR CF nail* OR CFR nail* OR Carbon nail* OR PEEK nail* OR radiolucent nail*)

## References

[1] Nilssen P., McKelvey K., Lin C. (2024). Revision Surgery Risk After Open Reduction and Internal Fixation Versus Acute Total Hip Arthroplasty in Geriatric Acetabular Fractures: A Nationwide Study. JAAOS—J. Am. Acad. Orthop. Surg..

[2] Lunn K., Hurley E.T., Adu-Kwarteng K., Welch J.M., Levin J.M., Anakwenze O., Boachie-Adjei Y., Klifto C.S. (2025). Complications Following Intramedullary Nailing of Proximal Humerus and Humeral Shaft Fractures: A Systematic Review. J. Shoulder Elb. Surg..

[3] Sandhu M., Kumar N., Singh Sawhney R., Kaur S., Singh K. (2024). Materials Used in Orthopedic Implants: A Comprehensive Review Study. Obstet. Gynaecol. Forum.

[4] Shaikh M., Kahwash F., Lu Z., Alkhreisat M., Mohammad A., Shyha I. (2024). Revolutionising Orthopaedic Implants—A Comprehensive Review on Metal 3D Printing with Materials, Design Strategies, Manufacturing Technologies, and Post-Process Machining Advancements. Int. J. Adv. Manuf. Technol..

[5] Edelstein Y., Ohm H., Rosen Y. (2011). Metallosis and Pseudotumor after Failed ORIF of a Humeral Fracture. Bull. NYU Hosp. Jt. Dis..

[6] Steven M. (2019). Kurtz PEEK Biomaterials Handbook.

[7] Baba K., Mori Y., Chiba D., Kuwahara Y., Kurishima H., Tanaka H., Kogure A., Kamimura M., Yamada N., Ohtsu S. (2023). TiNbSn Stems with Gradient Changes of Young’s Modulus and Stiffness Reduce Stress Shielding Compared to the Standard Fit-and-Fill Stems. Eur. J. Med. Res..

[8] Callister W.D. (2006). Materials Science and Engineering: An Introduction.

[9] Zhang S., Patel D., Brady M., Gambill S., Theivendran K., Deshmukh S., Swadener J., Junaid S., Leslie L.J. (2022). Experimental Testing of Fracture Fixation Plates: A Review. Proc. Inst. Mech. Eng. Part H J. Eng. Med..

[10] Rahmitasari F., Ishida Y., Kurahashi K., Matsuda T., Watanabe M., Ichikawa T. (2017). PEEK with Reinforced Materials and Modifications for Dental Implant Applications. Dent. J..

[11] Li C.S., Vannabouathong C., Sprague S., Bhandari M. (2015). The Use of Carbon-Fiber-Reinforced (CFR) PEEK Material in Orthopedic Implants: A Systematic Review. Clin. Med. Insights Arthritis Musculoskelet. Disord..

[12] Page M.J., McKenzie J.E., Bossuyt P.M., Boutron I., Hoffmann T.C., Mulrow C.D., Shamseer L., Tetzlaff J.M., Akl E.A., Brennan S.E. (2021). The PRISMA 2020 Statement: An Updated Guideline for Reporting Systematic Reviews. Br. Med. J..

[13] Sterne J.A.C., Hernán M.A., Reeves B.C., Savović J., Berkman N.D., Viswanathan M., Henry D., Altman D.G., Ansari M.T., Boutron I. (2016). ROBINS-I: A Tool for Assessing Risk of Bias in Non-Randomised Studies of Interventions. Br. Med. J..

[14] Patton M.S., Lyon T.D.B., Ashcroft G.P. (2008). Levels of Systemic Metal Ions in Patients with Intramedullary Nails. Acta Orthop..

[15] Tanoğlu O., Say F., Yücens M., Alemdaroğlu K.B., İltar S., Aydoğan N.H. (2020). Titanium Alloy Intramedullary Nails and Plates Affect Serum Metal Ion Levels within the Fracture Healing Period. Biol. Trace Elem. Res..

[16] Jones D.M., Marsh J.L., Nepola J.V., Jacobs J.J., Skipor A.K., Urban R.M., Gilbert J.L., Buckwalter J.A. (2001). Focal Osteolysis at the Junctions of a Modular Stainless-Steel Femoral Intramedullary Nail. J. Bone Jt. Surg. Am. Vol..

[17] Cotic M., Vogt S., Hinterwimmer S., Feucht M.J., Slotta-Huspenina J., Schuster T., Imhoff A.B. (2015). A Matched-Pair Comparison of Two Different Locking Plates for Valgus-Producing Medial Open-Wedge High Tibial Osteotomy: Peek–Carbon Composite Plate versus Titanium Plate. Knee Surg. Sports Traumatol. Arthrosc..

[18] Krischak G.D., Gebhard F., Mohr W., Krivan V., Ignatius A., Beck A., Wachter N.J., Reuter P., Arand M., Kinzl L. (2004). Difference in Metallic Wear Distribution Released from Commercially Pure Titanium Compared with Stainless Steel Plates. Arch. Orthop. Trauma Surg..

[19] Park S.J., Bae G.C., Kwon D.G. (2022). Metallosis after Using Distal Fibular Locking Plate for Lateral Malleolar Fractures: A Retrospective Study. Arch. Orthop. Trauma Surg..

[20] Shahgaldi B.F., Compson J. (2000). Wear and Corrosion of Sliding Counterparts of Stainless-Steel Hip Screw-Plates. Injury.

[21] Yeo E.D., Kim H.J., Cho W.I., Lee Y.K. (2015). A Specialized Fibular Locking Plate for Lateral Malleolar Fractures. J. Foot Ankle Surg..

[22] Foong B., Panagiotopoulou V.C., Hothi H.S., Henckel J., Calder P.R., Goodier D.W., Hart A.J. (2018). Assessment of Material Loss of Retrieved Magnetically Controlled Implants for Limb Lengthening. Proc. Inst. Mech. Eng. H..

[23] Law G.W., Koh J.S.B., Yew A.K.S., Howe T.S. (2020). Scanning Electron Microscopy Study of Retrieved Implants Suggests a Ratcheting Mechanism behind Medial Migration in Cephalomedullary Nailing of Hip Fractures. Malays. Orthop. J..

[24] Fleischhacker E., Sprecher C.M., Milz S., Saller M.M., Wirz R., Zboray R., Parrilli A., Gleich J., Siebenbürger G., Böcker W. (2024). Inflammatory Tissue Response in Human Soft Tissue Is Caused by a Higher Particle Load near Carbon Fiber-Reinforced PEEK Compared to Titanium Plates. Acta Biomater..

[25] Bertoldi C., Zaffe D., Bellini P., Consolo U. (2001). Metallic Elements in Tissues Surrounding Internal Rigid Fixation (IRF) Devices. Minerva Stomatol..

[26] Kumar A., Zaidi S.M.H., Sahito B., Kumar D., Ali M. (2020). A Case Report of Metallosis with a Failed Distal Femur Plate. Cureus.

[27] Merolli A., Rocchi L., De Spirito M., Federico F., Morini A., Mingarelli L., Fanfani F. (2016). Debris of Carbon-Fibers Originated from a CFRP (PEEK) Wrist-Plate Triggered a Destruent Synovitis in Human. J. Mater. Sci. Mater. Med..

[28] Metikala S., Bhogadi P. (2015). Orthogonal Double Plating and Autologous Bone Grafting of Postoperative Humeral Shaft Nonunion—A Rare Case Report and Review of Literature. Orthpaedic Case Rep. J. Orthop. Case Rep..

[29] Tang C.Q.Y., Chuah K.L., Teoh L.C. (2023). Metallosis Following Titanium Implant Use in the Hand: A Case Report and Review of Current Literature. J. Hand Microsurg..

[30] Kang R., Stern P.J. (2002). Humeral Nonunion Associated with Metallosis Secondary to Use of a Titanium Flexible Humeral Intramedullary Nail: A Case Report. J. Bone Jt. Surg. Am. Vol..

[31] Ngo D., Todd M., Accadbled F., Foster B., Jellesen M.S., Rölfing J.D., Rawat J. (2024). Corrosion of a Fassier-Duval Telescopic Nail Causing Pain and Osteolysis: A Case Report. JBJS Case Connect.

[32] Bozic K.J., Browne J., Dangles C.J., Manner P.A., Yates A.J.J., Weber K.L., Boyer K.M., Zemaitis P., Woznica A., Turkelson C.M. (2012). Modern Metal-on-Metal Hip Implants. JAAOS—J. Am. Acad. Orthop. Surg..

[33] Stratton-Powell A.A., Pasko K.M., Brockett C.L., Tipper J.L. (2016). The Biologic Response to Polyetheretherketone (PEEK) Wear Particles in Total Joint Replacement: A Systematic Review. Clin. Orthop. Relat. Res..

[34] Jacobs J.J., Skipor A.K., Patterson L.M., Hallab N.J., Paprosky W.G., Black J., Galante J.O. (1998). Metal Release in Patients Who Have Had a Primary Total Hip Arthroplasty. A Prospective, Controlled, Longitudinal Study. J. Bone Jt. Surg..

[35] Vertesich K., Staats K., Böhler C., Koza R., Lass R., Giurea A. (2022). Long Term Results of a Rotating Hinge Total Knee Prosthesis with Carbon-Fiber Reinforced Poly-Ether-Ether-Ketone (CFR-PEEK) as Bearing Material. Front. Bioeng. Biotechnol..

